# Awareness and Attitude Regarding Contact Lens Use Among Female Students at the University of Bisha: A Cross-Sectional Study

**DOI:** 10.7759/cureus.58216

**Published:** 2024-04-13

**Authors:** Yousef Al-Otaibi, Abdulmajeed Alkhathami, Rana Saad Alojair, Mohammed A Alharthi, Yazeed Alshahrani, Sara Saeed Alaklabi, Masoud M Alqahtani, Amaal M Almalki, Ashwaq Y Asiri

**Affiliations:** 1 Department of Ophthalmology, Faculty of Medicine, University of Bisha, Bisha, SAU; 2 Department of Ophthalmology, Armed Forced Hospital Southern Region, Khamis Mushait, SAU; 3 College of Medicine, Faculty of Medicine, University of Bisha, Bisha, SAU; 4 Department of Internal Medicine, Faculty of Medicine, University of Bisha, Bisha, SAU; 5 Department of Emergency Medicine, Faculty of Medicine, University of Bisha, Bisha, SAU; 6 Department of Ophthalmology, King Abdulaziz Specialist Hospital, Taif, SAU; 7 Department of Ophthalmology, College of Medicine, King Khalid University, Abha, SAU

**Keywords:** eye care, female college students, contact lens, attitude, awareness

## Abstract

Introduction: Given the growing popularity of contact lenses among female students in Saudi Arabia for various reasons including vision correction as well as aesthetic and therapeutic purposes, it is essential to understand the knowledge about them, their uses, and how to handle them, given their significance in healthcare.

Methods: A cross-sectional study design was utilized in this research, employing data from a sample of 413 female students at the University of Bisha. The participants completed a self-administered questionnaire and were ensured anonymity.

Results: The study results showed that 52.9% (n=218) had a good knowledge level while 47.1% (n=195) had poor knowledge about contact lenses use. The results established a statistically significant association between region, faculty, and study year (p<0.005) with p-values of 0.002, 0.001, and 0.005, respectively, and level of knowledge about contact lens use. There was no statistically significant association between the age of the participants and the level of knowledge about contact lens use.

Conclusion: Although there was a generally good level of awareness about contact lens use among female students, there is still insufficiency in knowledge with regard to particular aspects such as cleaning the contact lenses and contact lens cover. The study finds the need for more ocular educational sessions with ophthalmologists to increase awareness about contact lenses.

## Introduction

The popularity of contact lenses is growing globally, with more people choosing them for vision correction. They have various uses, including therapeutic, aesthetic, and corrective purposes. Refractive errors are becoming more common, increasing reliance on contact lenses [1]. Recognizing the levels of awareness is crucial, given that contact lenses are medical devices. Most contact lens wearers believe that getting an eye exam before using lenses is essential and that regular exams are also crucial [2]. Typically, potential side effects of wearing lenses are mild, but they can occasionally pose serious risks to vision. Educational initiatives are needed to increase awareness, especially among different age groups and demographics. The study by Leeamornsiri and Titawattanakul has shown that medical students had better knowledge of CL care and use than non-medical students [3]. Lee et al. reported that more urgent care cases related to CL were because fo extended wear [4]. Public education is necessary to reduce eye infections and other issues related to contact lens use.

People from more privileged backgrounds tend to be more accepting of those who wear contact lenses, suggesting that social class shapes attitudes towards this technology. It is thought to result from a self-selection process whereby financially stable people are more inclined to choose contact lenses to correct their vision. The inclination for financially stable individuals to opt for contact lenses over traditional eyeglasses can be attributed to several factors. Contact lenses often require regular purchases, such as replacements and maintenance solutions, which can be costlier than a one-time investment in eyeglasses. Individuals with stable finances may find it easier to afford these ongoing expenses associated with contact lenses [5].

It is important to use soft CLs responsibly and take care of them after each use to reduce the risk of microbial keratitis (MK) [6]. Many students say they learned how to put on and take off CLs from their eye doctors; most of these students cite visual correction for refractive errors as the main reason they use CLs [7]. In a study by Schein et al., more than half of the users (57%) reported using CLs for over two years [8]. A previous study discovered a correlation between people's hand-washing behaviors and the removal of contact lenses [7]. This highlights the potential risk of bacterial and fungal keratitis, which are eye infections, that can arise due to insufficient hand-washing practices. There is a spectrum of practices among wearers of contact lenses, with 61.9% reporting never or rarely sharing of the contact lens with others and 19.2% admitting to frequent sharing [9].

The adoption of contact lenses (CL) as a popular vision correction option has surged in recent years, particularly among female students [10]. With the increasing prevalence of contact lens use among this demographic, understanding the level of awareness and attitudes towards their usage becomes paramount. As medical devices, contact lenses require proper handling and care to ensure optimal eye health. However, there remains a gap in knowledge regarding their correct usage and maintenance, especially among younger populations. This study aims to delve into the intricacies of contact lens usage among female students at the University of Bisha, Saudi Arabia. By conducting a cross-sectional study, this research seeks to assess the knowledge levels, usage patterns, and attitudes toward contact lenses among female students. The University of Bisha serves as an ideal setting for this study, given its diverse student population and the potential impact of cultural and environmental factors on contact lens practices. Understanding the awareness and attitudes with regard to CL among female students can inform targeted educational interventions aimed at promoting safe and responsible CL practices. Moreover, insights from this study can contribute to enhancing eye health awareness and improving the overall well-being of this demographic.

## Materials and methods

This was a cross-sectional study conducted at the University of Bisha, Bisha, Saudi Arabia, to evaluate awareness and attitude with regard to CL among female students. All female students who agreed and gave consent to participate in the study were included and male students and female students from other universities were excluded.

Sample size 

Researchers determined the required sample size using a standard formula for cross-sectional studies: (n0= (z2pq)/ d2), where n0=Minimum sample size, Z= standard normal deviation (1.96), p=population proportion (0.65), q= (1-p) =0.35, and d=margin of error (0.05).

The necessary sample size for this cross-sectional study was determined based on standard statistical principles. Following calculation, the study initially aimed for a sample size of 384 female students. To reduce potential bias, an additional 7.5% was included, resulting in a final sample size of 413 participants.

Sampling technique

The study employed a simple random sampling technique to select participants based on their accessibility and willingness to participate from various faculties and academic levels at the University of Bisha.

Data collection

Data were collected through a web-based validated questionnaire from the participants. The questionnaire consisted of demographic information (e.g., age, region, faculty, and study year) and questions related to awareness and attitudes regarding CL use. 

Data analysis 

After collecting the questionnaires, researchers entered the data into IBM SPSS Statistics for Windows, Version 25.0 (Released 2017; IBM Corp., Armonk, New York, United States). They use descriptive statistics such as frequencies, percentages, means, and standard deviations to summarize the demographic information and responses related to awareness and attitude toward CL use among female students at the University of Bisha. They also employed inferential statistics, including chi-square tests to examine associations between demographic variables and awareness levels.

Ethical considerations

The study adheres to ethical considerations such as obtaining informed consent from participants, ensuring confidentiality and anonymity, and respecting participants' rights to withdraw from the study at any time. The study adhered to the World Medical Association (WMA) Declaration of Helsinki: Ethical Principles for Medical Research Involving Human Subjects, as amended at the 59th WMA General Assembly in Seoul, Republic of Korea. No information that might be used to identify the participants personally was gathered. In addition, the Research Ethics Committee at King Abdullah Hospital (E-CTS) reviewed and agreed on this project (REF No. BIS-24-00006-27032024).

## Results

A total of 413 female students of the University of Bisha completed the questionnaires. The majority (65.4%, n=270) of the participants were aged 18-21 years with more than half (64.2%, n=256) being residents of urban areas (Table 1). The College of Medicine contributed 21.5% (n=89) of the participants, while the College of Applied Medical Sciences provided 15.7% (n=65) of the participants.

**Table 1:** Socio-demographic information of the participants (N=413) Data are presented as frequency (n) and proportion (%)

Socio-demographic information	Category	Frequency (Proportion)
Age (years)	18-21	270 (65.4%)
	22-25	104 (25.2%)
	26-29	20 (4.8%)
	Older than 30	19 (4.6%)
Region	Urban area	265 (64.2%)
	Rural area	148(35.8%)
Faculty	College of Applied Medical Sciences	65 (15.7%)
	College of Business	47 (11.4%)
	College of Computer and Information and Technology	57 (13.8%)
	College of Engineering	20 (4.8%)
	College of Home Economics	16 (3.9%)
	College of Medicine	89 (21.5%)
	College of Science	33 (8.0%)
	College of Science and Literature	56 (13.6%)
	Others	30 (7.3%)
Study year	First year	66 (16.0%)
	Second year	125 (30.3%)
	Third year	86 (20.8%)
	Fourth year	93 (22.5%)
	Fifth year	13 (3.1%)
	Sixth year	22 (5.3%)
	Others	8(2.0%)

Table 2 depicts the attitude regarding and practices of CL use among the female students at the University of Bisha. The findings demonstrate that a substantial proportion (43.8%, n=181) of the participants used CL; with more than half (52.5%, n=95) of these having used CLs for a period of less than six months, 45.9% (n=83) of the users used CLs for cosmetic purposes, while 32.0% (n=58) used them for medical purposes. A large proportion (46.4%, n=84) of the respondents who used CLs, used disposable CLs with the vast majority (78.0%, n=141) using them occasionally. The majority (70.2%, n=127) of the female students who used CLs, used them for less than eight hours per day. 

**Table 2:** Participants’ attitudes and practices with regard to contact lens use Data are presented as frequency (n) and proportion (%)

Questions	Categories	Frequency (Proportion)
Do you use contact lens (N=413)	Yes	181 (43.8%)
	No	232 (56.2%)
Why do you use the contact lens (N=181)	Medical purpose	58 (32.0%)
	Cosmetic purpose	83 (45.9%)
	Both	40 (22.1%)
Type of contact lens (N=181)	Daily wear	76 (42.0%)
	Disposable	84 (46.4%)
	Extended wear	21 (11.6%)
Use of contact lens (N=181)	Continuous	40 (22.0%)
	Occasional	141 (78.0%)
How many hours per day do you use the contact lens (N=181)	Less than 8 hours	127 (70.2%)
	8-12 hours	43 (23.8%)
	More than 12 hours	11 (6.0%)
If you use contact lens, for how long have you been using them (N=181)	Less than 6 months	95 (52.5%)
	6-12 months	41 (22.7%
	1-5 years	27 (14.9%)
	5 years or more	18 (9.9%

**Figure 1:**
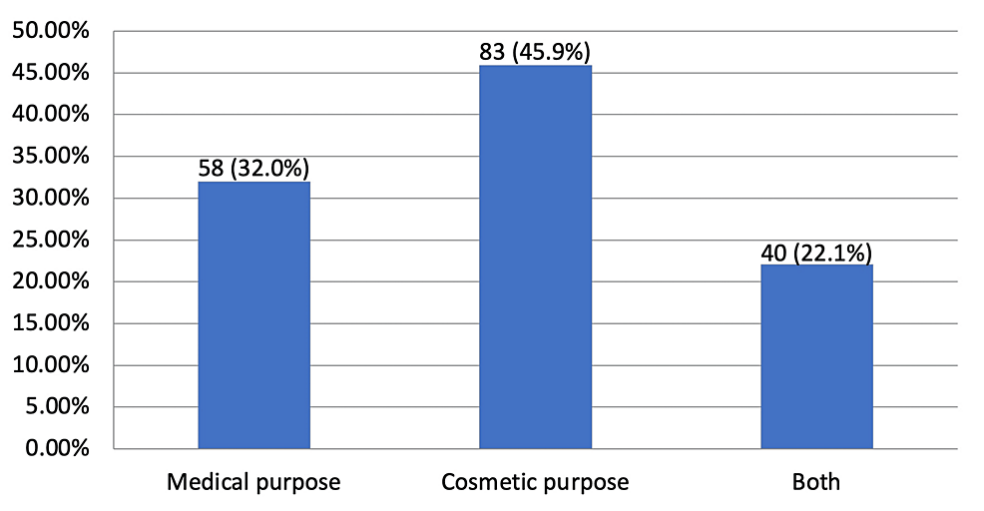
Distribution of contact lens use among the students who used them (N=181) Data are presented as frequency (n) and proportion (%)

Figure 1 depicts the distribution of CL uses among the female students at the University of Bisha who used CLs. The results noted that the majority (n=83, 45.9%) of the users used CLs for cosmetic purposes while 58 (32.0%) used them for medical purposes; 40 (22.1%) student users used CLs for both medical and cosmetic purposes.

Table 3 presents information on the general awareness of CL. Only 10.8% (n=44) of the female students were aware that washing CLs before wearing them is not necessary. Fifteen (3.7%) were aware that washing the lens cover is not necessary as well. The vast majority (93.2%, n=385) of the respondents strongly agreed and agreed that hand washing when using CLs is necessary. More than half (72.1%, n=298) of the respondents knew that it is necessary to routinely visit an ophthalmologist for CL check-up. Furthermore, 72.5% (n=300) of the respondents correctly indicated that changing the CLs depends on the comfort level.

**Table 3:** Participants’ general awareness about contact lenses Data are presented as frequency (n) and proportion (%)

Statement	Strongly agree, n (%)	Agree, n (%)	Neutral, n (%)	Disagree, n (%)	Strongly disagree, n (%)
Do you think that washing the lens with saline solution before wearing is necessary?	154 (37.2%)	146 (35.3%)	69(16.7%)	31 (7.5%)	13 (3.3%)
Do you think washing the lens container is necessary?	183 (44.3%)	171 (41.4%)	44 (10.6%)	13 (3.3%)	2 (0.4%)
Do you think hand washing before applying the Lens is necessary?	254 (61.5%)	131 (31.7%)	23 (5.7%	4 (0.9%)	1 (0.2%
Do you think routine visits to ophthalmologists as follow-up is necessary?	124 (30.0%)	174 (42.1%)	97 (23.4%	18 (4.5%)	-
Does the decision to change the contact lens depend on comfort level or specific time?	109 (26.3%)	191 (46.2%)	70 (16.9%)	32 (7.9%)	11 (2.7%)

Table 4 depicts the relationship between participants’ demographics such as age, region, faculty, study year, and level of knowledge about CL use. The results established a statistically significant association of region, faculty, and study year with the level of knowledge about CL use with p-values<0.005 (0.002, 0.001, and 0.005, respectively). No statistically significant association was found between age and the level of knowledge about CL use. 

**Table 4:** The association between age, region, faculty, and study year and level of knowledge about contact lens use Data are presented as frequency (n) and proportion (%) * Significant at p<0.05 level.

	Level of knowledge			
Variables	Category	Poor - n(%)	Good - n(%)	p value
Age	18-21	116 (42.7%)	154 (57.3%)	0.254
	22-25	49 (46.9%)	55 (53.1%)	
	26-29	9 (44.3%)	11 (55.7%)	
	Older than 30	9 (47.4%)	10 (52.6%)	
Region	Urban area	107 (40.2%)	158 (59.8%)	0.002
	Rural area	78 (52.5%)	70 (47.5%)	
Faculty	College of Applied Medical Sciences	24 (37.6%)	41 (62.4%)	0.001
	College of Business	25 (53.7%)	22 (46.3%)	
	College of Computer and Information and Technology	31 (54.9%)	26 (45.1%)	
	College of Engineering	11 (55.0%)	9 (45.0%)	
	College of Home Economics	9 (55.7%)	7 (44.3%)	
	College of Medicine	33 (36.8%)	56 (63.2%)	
	College of Science	14(43.1%)	19 (56.9%)	
	College of Science and Literature	27 (47.7%)	29 (52.3%)	
	Others	18 (60.1%)	12 (39.9%)	
Study year	First year	35 (53.3%)	31 (46.7%)	0.005
	Second year	60 (41.8%)	65 (58.2%)	
	Third year	35 (41.1%)	51 (58.9%)	
	Forth year	38 (40.9%)	55 (59.1%)	
	Fifth year	5 (40.2%)	8 (59.8%)	
	Sixth year	9 (39.8%)	13 (60.2%)	
	Others	5 (59.9%)	3 (40.1%)	

**Figure 2:**
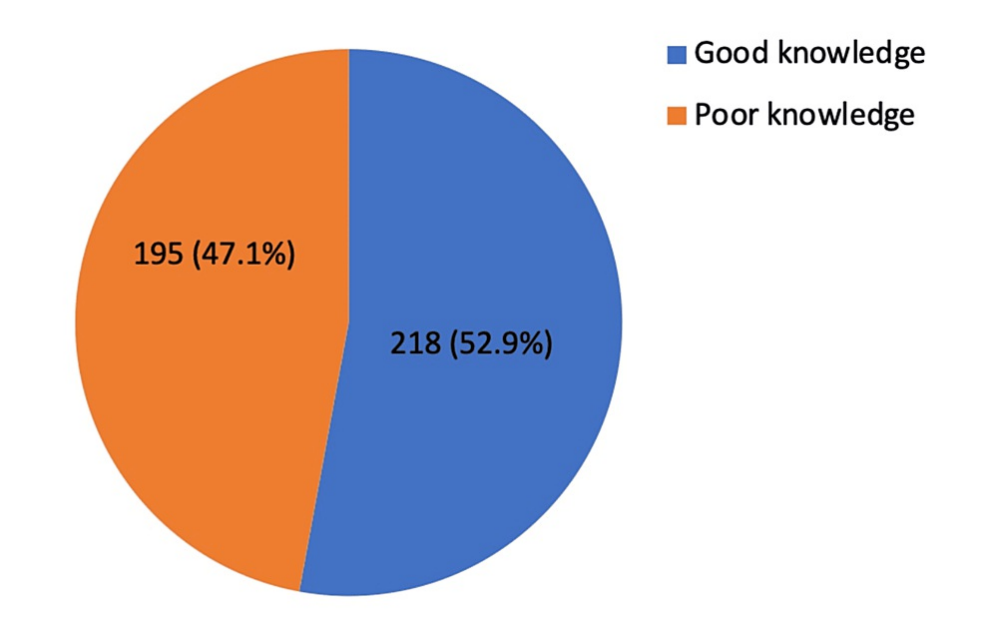
Distribution of good knowledge and poor knowledge about contact lens use.

Figure 2 illustrates how knowledge is distributed in terms of good knowledge and poor knowledge among female students of the University of Bisha. The study results show that 52.9% (n=218) had a good knowledge level while 47.1% (n=195) had poor knowledge about CL use.

## Discussion

The study aimed to evaluate the awareness and attitude regarding CL use among female students at the University of Bisha. The sample for the current study primarily consisted of female students aged 18-21 years, the majority of them were residents of urban areas and from the College of Medicine and College of Applied Medical Science. 

The findings revealed that 52.9% (n=218) of the female students had good knowledge about the use of CL. The findings concur with those of the study conducted by Alanazi and Alanazi in Saudi Arabia which showed that 64% of the female students at Northern Border University had sufficient knowledge about the use of contact lenses [11]. The study showed that 59.8% (n=158) of the female students who were residents of urban areas had excellent levels of knowledge about CL. These findings mirror those of a study by Alobaidan et al., which revealed that 54.7% of Saudi urban CL users had sufficient knowledge about CL use and care [12].

A study conducted by Ibrahim et al. in Jeddah, Saudi Arabia, revealed that 50.2% of the medical students from King Abdulaziz University had used CL at least once during their lifetime [13]. The current study, by including students from various colleges of the University, has observed a slightly higher proportion of CL users compared to a previous study [14]. This suggests that the diversity of participants from different academic backgrounds may have contributed to a more comprehensive representation of CL usage patterns within the university community. A substantial proportion (45.9%, n=83) of CL users in the current study used CL for cosmetic purposes while a few (32.0%, n=58) used them for medical purposes. The findings are consistent with the findings of the study conducted by Wongkrajang and Neeser which revealed that the majority of female students from Chulalongkorn University who used CL, used them for cosmetic purposes [15].

Although the majority of the female students demonstrated adequate knowledge about CL use, only 10.8% (n=44) of them were aware that washing the lenses before wearing them is not necessary. The findings are in congruence with the study conducted by Alswailmi which noted a higher level of knowledge of CL use among female students at Hafr Al-Batin University; however, 51.3% of the students incorrectly believed that contact lenses can be cleaned with water [16]. Most of the participants (93.2%, n=385) showed good knowledge regarding hand hygiene when using contact lenses. The findings concur with those of a study conducted in Saudi Arabia by Alasiri et al. involving female medical students; the study noted that the medical students had sufficient knowledge with respect to hygiene and care when handling contact lenses [17]. The majority (72.1%, n=298) of the female students were aware of the need for routine visits to an ophthalmologist for CL check-ups. The findings are similar to those of a study conducted by Şengör et al. in Turkey which revealed that the majority of the CL users sought consultation from ophthalmologists [18]. Moreover, the current study noted that the majority (72.5%, n=300) of the female students correctly indicated that changing the CL depends on their comfort level. This was in line with Janti et al.'s assertion that medical college students in Tamil Nadu, India, would only change CL if they found them uncomfortable and not tolerated [19].

The current study established a statistically significant association between region, faculty, and study year and level of knowledge about CL use. However, there was no association between the age of the participants and the level of knowledge about CL use.

The major limitation of the study was the utilization of a cross-sectional study design, which can only identify relationships between components but not causalities. As this was a survey-based study, recollection bias might be a limitation that also needs further investigation. Additionally, considering that the study involved the administration of an online questionnaire, the study relied on respondents accurately documenting their responses without the ability to check this, which may have contributed to a potential bias.

## Conclusions

Overall, there was good knowledge and attitude regarding CL use among female students at the University of Bisha. Female students who were residing in urban areas and those from the College of Medicine and College of Applied Medical Sciences demonstrated more knowledge regarding CL use. However, there is still an insufficiency of knowledge and awareness in regard to particular aspects such as cleaning CLs and CL cases. The study finds the need for more ocular educational sessions with ophthalmologists to increase awareness about CL among students and the general public. 
